# Homogeneous Inflammatory Gene Profiles Induced in Human Dermal Fibroblasts in Response to the Three Main Species of *Borrelia burgdorferi* sensu lato

**DOI:** 10.1371/journal.pone.0164117

**Published:** 2016-10-05

**Authors:** Mariam Meddeb, Wassila Carpentier, Nicolas Cagnard, Sophie Nadaud, Antoine Grillon, Cathy Barthel, Sylvie Josiane De Martino, Benoît Jaulhac, Nathalie Boulanger, Frédéric Schramm

**Affiliations:** 1 EA7290 Early Bacterial Virulence: Lyme borreliosis Group, FMTS, Université de Strasbourg, Strasbourg, France; 2 Plate-forme Post-Génomique P3S, Université Pierre et Marie Curie, Faculty of Medicine, Paris, France; 3 Plateforme Bio-informatique, Université Paris Descartes, Structure Fédérative de Recherche Necker, INSERM US24/CNRS UMS 3633, Paris, France; 4 INSERM UMR 1166, Université Pierre et Marie Curie, Université Paris 06, Paris, France; 5 French National Reference Center for *Borrelia*, University Hospital, Strasbourg, France; University of Kentucky College of Medicine, UNITED STATES

## Abstract

In Lyme borreliosis, the skin is the key site for bacterial inoculation by the infected tick and for cutaneous manifestations. We previously showed that different strains of *Borrelia burgdorferi* sensu stricto isolated from tick and from different clinical stages of the Lyme borreliosis (erythema migrans, and acrodermatitis chronica atrophicans) elicited a very similar transcriptional response in normal human dermal fibroblasts. In this study, using whole transcriptome microarray chips, we aimed to compare the transcriptional response of normal human dermal fibroblasts stimulated by 3 *Borrelia burgdorferi* sensu lato strains belonging to 3 main pathogenic species (*B*. *afzelii*, *B*. *garinii* and *B*. *burgdorferi* sensu stricto) in order to determine whether “species-related” inflammatory pathways could be identified. The three *Borrelia* strains tested exhibited similar transcriptional profiles, and no species-specific fingerprint of transcriptional changes in fibroblasts was observed. Conversely, a common core of chemokines/cytokines (CCL2, CXCL1, CXCL2, CXCL6, CXCL10, IL-6, IL-8) and interferon-related genes was stimulated by all the 3 strains. Dermal fibroblasts appear to play a key role in the cutaneous infection with *Borrelia*, inducing a homogeneous inflammatory response, whichever *Borrelia* species was involved.

## Introduction

Lyme borreliosis is caused by spirochetes of the *Borrelia burgdorferi* sensu lato (sl) group that are transmitted by *Ixodes* spp. ticks: the bacteria are inoculated intradermally, multiply locally and eventually spread to various secondary organs throughout the body, especially the skin, the nervous system and the joints, inducing inflammatory lesions [1]. Lyme borreliosis is the most common tick-borne infectious disease in North America [2] and in countries with moderate climates in Eurasia [3]. Whereas *B*. *burgdorferi* sensu stricto (ss) is the only main *Borrelia* species responsible for Lyme borreliosis manifestations in North America, at least 8 species can be pathogenic for humans in Europe, with 5 clearly pathogenic species—*B*. *burgdorferi* ss, *B*. *afzelii*, *B*. *garinii*, *B*. *bavariensis* [4] and *B*. *spielmanii* [5]—, and 3 rarely, if at all, pathogenic species for humans—*B*. *bissettii* [6, 7], *B*. *lusitaniae* [8] and *B*. *valaisiana* [9]. The erythema migrans (EM), first clinical manifestation of Lyme borreliosis and considered the hallmark of the disease, can be caused by any pathogenic *Borrelia* species of the *B*. *burgdorferi* sl group. In contrast, acrodermatitis chronica atrophicans (ACA) is typically associated with *B*. *afzelii*, neurological manifestations most commonly involve *B*. *garinii* and arthritis is preferentially related to *B*. *burgdorferi* ss [10].

The skin is a key interface in Lyme borreliosis since it represents both the inoculation site of the spirochetes, a potential filter to select specific invasive clones injected at the tick-bite site [11, 12], and also the target of clinical manifestations at various stages of the disease [13]. However, factors determining the limitation of the disease to a benign localised EM or the appearance of systemic manifestations and the way the bacteria escape the local cutaneous immunity, spread, and reach distal target organs are unknown. Thus, understanding the interaction of the bacteria with skin cells is essential. The role of the skin’s immune cells in anti-*Borrelia* defense mechanisms was addressed in several previous works [14–16], but the skin’s resident cells (keratinocytes and fibroblasts) were also shown to be sensors of danger [13] and to play a key role in the anti-*Borrelia* response [17, 18]. In particular, we previously showed that *Borrelia* induces the transcription of numerous inflammatory genes in normal human dermal primary fibroblasts (NHDF) [19], including chemokines and cytokines and many transcription factors and components of the extracellular matrix. As the 3 *B*. *burgdorferi* ss strains tested in our model were isolated from different stages of the human infection and elicited very similar transcriptional modulation profiles, we concluded that *Borrelia* pathotype has little influence on the fibroblasts’ response. Since the 3 strains used in our previous study belonged to the same species, in this new study, we wanted to assess whether the species of *Borrelia* could influence the transcriptional modulations of fibroblasts. For this purpose, we tested 3 *Borrelia* strains, all isolated from erythema migrans lesions and belonging to 3 different pathogenic species (*B*. *afzelii*, *B*. *garinii* and *B*. *burgdorferi* ss) in our NHDF *in vitro* model, and compared the transcriptional profiles induced by the different species using whole transcriptome microarrays.

## Materials and Methods

### Spirochetes strains

Three different strains of *Borrelia* representing the 3 major European species were chosen among the collection of clinical isolates maintained in the Institute of Bacteriology of Strasbourg: *B*. *afzelii* strain IBS17, *B*. *garinii* strain IBS6 and *B*. *burgdorferi* ss strain IBS19. These 3 strains were isolated from patients presenting a solitary EM as a unique clinical manifestation (Table 1). Each strain was used at passage ≤ 7, cultured in BSK-H medium (Sigma, Saint Quentin Fallavier, France) at 33°C with 5% CO_2_, and washed twice before the assays as previously described [19]. Careful washing of *Borrelia* prior to every coincubation experiment was a prerequisite before every stimulation experiment since, in our preliminary experiments, we observed that the BSK medium by itself induces a nonspecific transcriptional response in human dermal fibroblasts that was significantly higher than that of negative control fibroblasts (data not shown).

**Table 1:** Origin of the *Borrelia* strains used in this study.

n°	Species	Geographical origin, Country	Anatomical origin	Associated symptoms
IBS6	*B*. *garinii*	Strasbourg, France	Skin biopsy of EM^a^	none
IBS17	*B*. *afzelii*	La Roche Guyon, France	Skin biopsy of EM	none
IBS19	*B*. *burgdorferi* ss	Metz, France	Skin biopsy of EM	none

^a^ Erythema migrans.

### Fibroblast culture and stimulation

Primary human dermal fibroblasts (NHDF, Promocell, Heidelberg, Germany) were maintained in FGM2 medium (detailed characteristics of the fibroblast’s batches are presented in Table 2). To stimulate the cells, fibroblasts were used at passage 5 and seeded at 7.5 x 10^4^ per well in a 24-well plate. At confluence and one day before *Borrelia* activation, FGM2 medium was replaced by FGM medium without fetal calf serum. If not otherwise stated, fibroblasts were stimulated with *B*. *burgdorferi* spirochetes at a multiplicity of infection (MOI) of 100:1 (100 *Borrelia* per fibroblast) for 24 hours.

**Table 2:** Characteristics of the fibroblast batches used in this study.

NHDF	Batch n°	Anatomical origin	Age^a^	Sexe	Race
#1	3021904	breast	20	female	caucasian
#2	3022702	eyelid	68	female	caucasian
#3	10118012	temple	70	male	caucasian
#4	10215061	cheek	56	female	caucasian
#5	20612062	cheek	60	male	caucasian
#6	70711061	lip	32	female	caucasian

^a^ Age is given in years.

In order to provide evidence that our experimental methods and specifically the coincubation of *Borrelia* and fibroblasts in a BSK-free medium was compatible with the viability of *Borrelia*, viability experiments were conducted: *Borrelia* were washed twice, suspended in FGM medium and either incubated as is (at a concentration of 2.5 x 10^6^ *Borrelia* per ml), or coincubated with fibroblasts at a MOI of 100:1. Twenty-four hours later, the culture medium (consisting of either *Borrelia* in FGM medium or *Borrelia* in FGM medium coincubated with human dermal fibroblasts), was recollected and instilled in BSK medium. We observed that both conditions provided viable *Borrelia* 96 hours later (data not shown).

### IL-8 ELISA

IL-8 secretion levels were measured in culture supernatants of unstimulated and *Borrelia*-stimulated cells by ELISA. The protocol was based on sandwich techniques, as described by the manufacturer (R&D systems, Lille, France). Experiments of cell stimulation with spirochetes were carried out twice in independent experiments. Results are presented as means ± standard deviations (SDs) of triplicate values and are representative of the two independent experiments.

### RNA extraction and semiquantitative real time RT-PCR

After removal of the supernatant, fibroblasts were directly resuspended in Trizol (Invitrogen, Cergy-Pontoise, France) and stored at -80°C until use. RNA extraction was performed with RN easy mini-kit (Qiagen, Courtaboeuf, France) according to the manufacturer’s protocol, including treatment with DNase (Ambion, Courtaboeuf, France). Two μg of total RNA were reverse-transcribed with the Superscript II first-strand synthesis system (Invitrogen, Cergy-Pontoise, France). Semiquantitative reverse transcription PCR (QRT-PCR) was done on an ABI Prism 7000 (Applied Biosystems, Courtaboeuf, France) with specific primers (S1 Table). Expression levels of all transcripts studied were normalised to housekeeping gene level and the relative changes in gene expression were compared with those of untreated cells using the 2^-ΔΔCt^ method. Two housekeeping genes were tested: β-actin and the RNA polymerase II genes [20].

### Microarray analysis

#### Sample generation and DNA microarray hybridisation and analysis

The design of the microarray experiment included 4 different analytical conditions: unstimulated fibroblasts, and fibroblasts stimulated with *B*. *burgdorferi* ss IBS19, *B*. *garinii* IBS6 and *B*. *afzelii* IBS17 strains at MOI 100:1 for 24h. For each analytical condition, a total of 6 biological replicates represented by 6 different batches of human dermal primary fibroblasts (Table 2) were used. A total of 24 biological samples was therefore processed for this microarray experiment.

The HumanHT-12 V4.0 expression BeadChip (Illumina Inc., Cambridge, UK), comprising 47,232 transcripts was used to generate gene expression profiles of unstimulated and *Borrelia*-stimulated fibroblasts. RNA was extracted using RNeasy Mini kit (Qiagen, Courtaboeuf, France). Each sample contained 50–200 ng RNA, and the quality was assessed with the 2100 Agilent Bioanalyzer. The cRNA was synthesized, amplified and purified using the Illumina TotalPrep RNA Amplification Kit (Ambion, Foster City, CA, USA) following manufacturer recommendations. Briefly, 200 ng of RNA was reverse transcribed. After second strand synthesis, the cDNA was transcribed *in vitro* and cRNA labelled with biotin-16-UTP. Labelled probe hybridisation to the Beadchips was carried out using Illumina’s protocol. The Beadchips were scanned on the Illumina IScan using Illumina IScan image data acquisition software. Illumina GenomeStudio software was used for preliminary data analysis, data normalisation and quality controls. Raw microarray intensity data was background subtracted and normalised using the “normalise quantiles” function. We used the detection P-values as flags, flag = 0, if P> 0.05 and flag = 1, if P≤ 0.05. Each tested probe list was created after filtering probes flagged ‘1’ for at least half of the chips involved in the comparison. The group comparisons were made using Student’s t-test. We filtered the resulting P-values at 5% and at a 2-fold threshold for functions and pathway analyses. For each particular sample, a given transcript was considered regulated if the mean fold change exceeded 1.2 in comparison with the “unstimulated” condition. The microarray data have been deposited in the GEO repository (http://www.ncbi.nlm.nih.gov/geo/) with the record number GSE77058. Microarray experiments were performed according to the MIAME guidelines [21].

## Results

### Human dermal fibroblasts stimulated by *B*. *burgdorferi* ss IBS19, *B*. *garinii* IBS6 and *B*. *afzelii* IBS17 secrete IL-8

**Fig 1:**
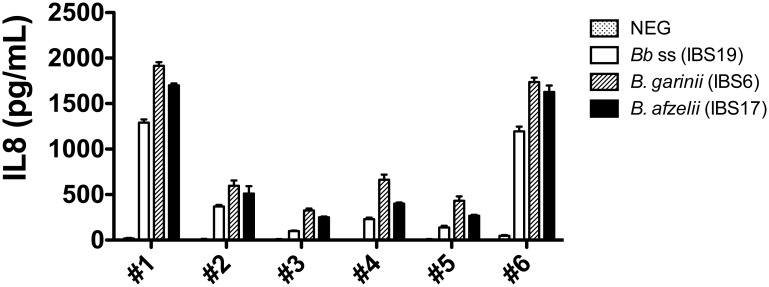
Measurement of IL-8 secretion by 6 different batches of fibroblasts coincubated with strains of 3 different species of the *B*. *burgdorferi* sl group. IL-8 secretion of fibroblasts stimulated by *B*. *burgdorferi* ss (*Bb* ss) IBS19, *B*. *garinii* IBS6, and *B*. *afzelii* IBS17 at MOI of 100:1 after 24h of stimulation. Each bar shows the mean ± SDs of triplicate values (technical replicates) obtained for each batch of fibroblasts—fibroblasts batch #1 to fibroblasts batch #6 (biological replicates). The results are representative of 2 independent experiments.

When coincubated with human primary dermal fibroblasts, different *B*. *burgdorferi* ss strains were shown to induce a pro-inflammatory response with IL-8 synthesis [18, 19]. To analyze the impact of the *Borrelia* species in our experimental model, we first measured the IL-8 synthesis by fibroblasts stimulated by *B*. *burgdorferi* ss IBS19, *B*. *garinii* IBS6 and *B*. *afzelii* IBS17. The 6 batches of fibroblasts tested in our study were able to produce the IL-8 chemokine. Even though absolute levels of IL-8 were highly variable between batches of cells tested, the levels of IL-8 synthesis obtained with the 3 *Borrelia* strains used for stimulation were comparable in each batch of fibroblasts (Fig 1).

**Fig 2:**
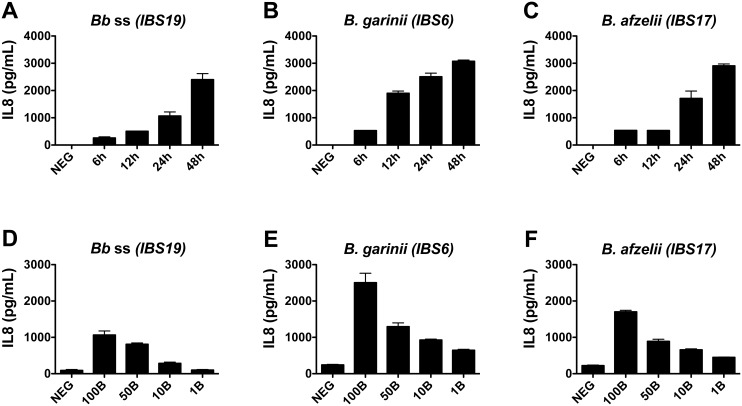
Kinetics and titration of IL-8 secretion by fibroblasts co-incubated with different species of the *B*. *burgdorferi* sl group. (A-C) Kinetic studies of IL-8 secretion of fibroblasts stimulated by *B*. *burgdorferi* ss (*Bb* ss) IBS19, *B*. *garinii* IBS6, and *B*. *afzelii* IBS17 at MOI of 100:1. (D-F) Levels of IL-8 secretion by fibroblasts stimulated by increasing concentrations (MOI of 1:1 = 1B, MOI of 10:1 = 10B, 50:1 = 50B, and 100:1 = 100B) of the 3 *Borrelia* strains at 24 hours. NEG: unstimulated fibroblasts. (A-F) Each bar shows the mean ± SDs of duplicate values obtained for fibroblasts batch #1.

A complete kinetic and titration study was performed for fibroblasts batch #1, and showed that the chemokine was secreted in a dose- and time-dependent manner for each of the 3 tested *Borrelia* strains (Fig 2). Similar profiles for titration experiments were obtained with fibroblasts expressing lower levels of IL-8 (S1 Fig).

### Global fibroblast transcriptional responses to *B*. *burgdorferi* ss IBS19, *B*. *garinii* IBS6 and *B*. *afzelii* IBS17

**Fig 3:**
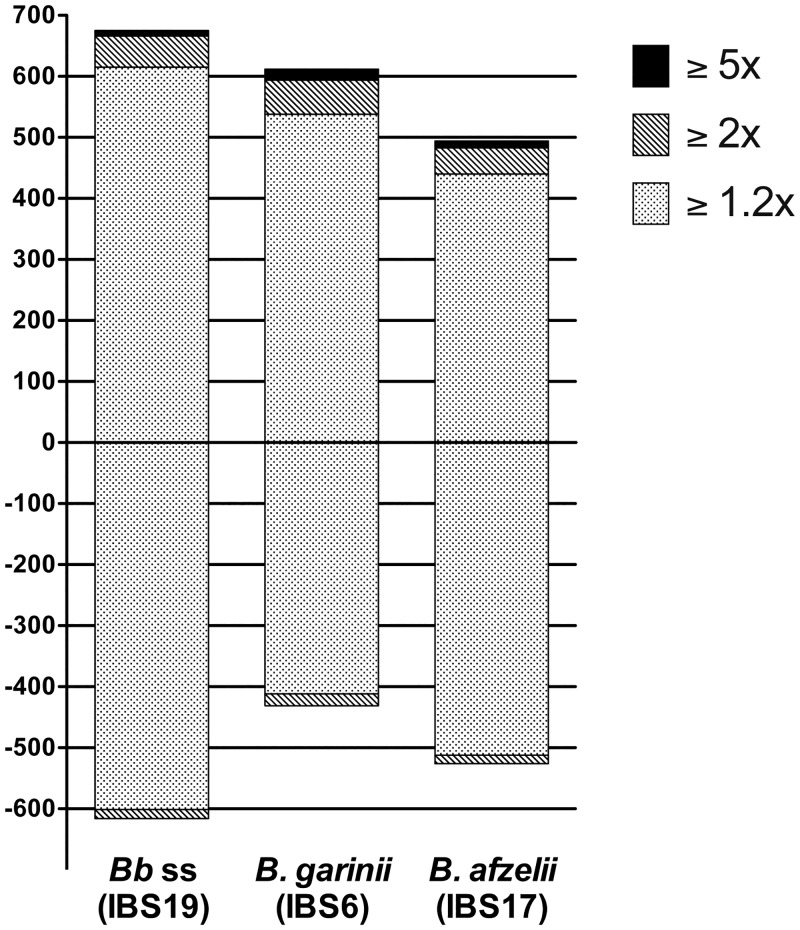
Gene expression profiles obtained from human fibroblasts stimulated with the three species of the *B*. *burgdorferi* sl group. Number of genes differentially expressed during fibroblast stimulation with *Borrelia*. The bars reflect the number of up-regulated genes (+) and down-regulated genes (-) for each strain. The light dotted areas correspond to gene expression changes of 1.2–2.0-fold, the gray hatched areas correspond to changes of 2.0–5.0-fold and black areas represent changes ≥5.0-fold.

Transcriptional modifications were studied by the Human HT12 BeadChip microarray, allowing whole transcriptome analysis. Statistical analysis was then performed after normalization of the raw results obtained for the 6 tested biological replicates (fibroblasts batch #1 to fibroblasts batch #6) and the 4 analytical conditions (unstimulated, *B*. *burgdorferi* ss IBS19-stimulated, *B*. *garinii* IBS6-stimulated and *B*. *afzelii* IBS17-stimulated) by comparing *Borrelia*-stimulated vs unstimulated samples. Out of 47,231 transcripts present on the chip, 1,968 transcripts (4.2%) were significantly regulated (≥1.2 fold vs unstimulated fibroblasts) by at least one the 3 tested strains. Among them, 1,291 transcripts were differentially regulated by *B*. *burgdorferi* ss IBS19 (675 up- and 616 down-regulated), 1,043 by *B*. *garinii* IBS6 (612 up- and 431 down-regulated), and 1,020 by *B*. *afzelii* IBS17 (494 up- and 526 down-regulated). The majority of these transcripts were regulated with a fold-change ranging from 1.2 to 2 for all 3 tested strains (Fig 3).

**Fig 4:**
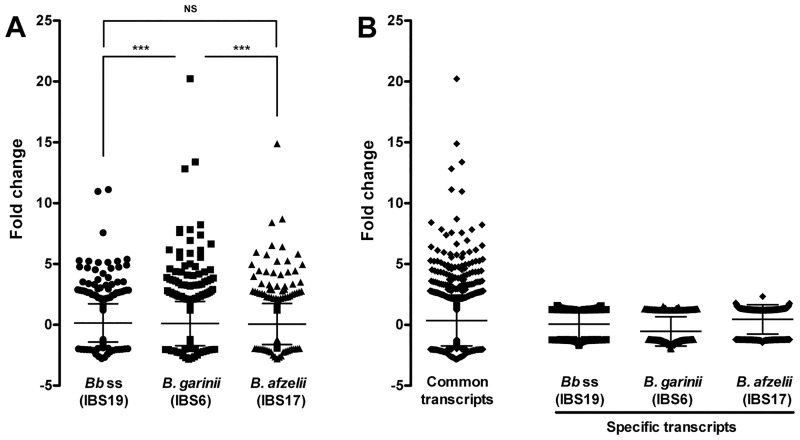
Intensity of transcriptional modifications. The distribution of regulated genes according to the level of regulation is expressed as fold-change in comparison to unstimulated cells. (A) Comparison of transcription levels between *Borrelia* strains. *** P <0.001, NS: not significant, tested by ANOVA using GraphPad Prism6 software (GraphPad, San Diego, CA). (B) Transcription levels of common (stimulated by the 3 strains) and specific (stimulated by only one of the strains) genes.

The global *Borrelia*-induced transcriptional modulation in fibroblasts was relatively similar in the 3 tested strains (similar number of up- and down-regulated genes) (Fig 3). Moreover, analyses of regulated genes showed that almost all the genes with high transcription modulation (≥2 fold vs unstimulated fibroblasts) were consistently stimulated by the 3 tested species (Fig 4B). The genes induced by only one of the tested species displayed mild transciptional modulations (between 1.2 and 2 fold vs unstimulated fibroblasts). However, it is noteworthy that the global level of modification induced by *B*. *garinii* IBS6 appeared significantly higher than that of *B*. *afzelii* IBS17 (P = 0.0007) and *B*. *burgdorferi* ss IBS19 (P = 0.0001), while the amplitude of activation induced by *B*. *afzelii* IBS17 and *B*. *burgdorferi* ss IBS19 did not statistically differ (Fig 4A).

### Highly up-regulated transcriptional responses are largely representative of pro-inflammatory pathways

We did not identify relevant specific strain-related transcriptional pathways. In contrast, we observed that the 3 strains belonging to 3 different species of the *B*. *burgdorferi* sl group elicited very similar transcriptional profiles in primary human dermal fibroblasts (see the global functional analyses and the signalisation pathway analyses results in S2 and S3 Figs). We then focused on co-regulated genes presenting the highest intensity of up-regulation. Among them, a common core of 42 genes were highly up-regulated in response to stimulation by all 3 *Borrelia* species. These mainly included pro-inflammatory genes involved in the innate immune response (Table 3). High levels of chemokines/cytokines were induced (CCL2, CXCL1, CXCL2, CXCL6, CXCL10, IL-6, IL-8), with fold-change values up to 20 for IL-8 in fibroblasts stimulated by *B*. *garinii* IBS6, along with a large number of genes representative of intracellular signalling pathways that sustain inflammatory responses, such as NF-κB- and interferon (IFN)-related genes. Whichever strain we tested, the IFN signalling and the activation of IRF by cytosolic pattern recognition receptors were found to be the two most overexpressed pathways in the signalisation pathway analyses (S2 Fig). Several other genes related to the metabolism, such as SOD2, were up-regulated by all 3 *Borrelia* species. Among all these genes, 7 of them (CXCL1, IL-6, IL-8, OAS2, STAT1, IFIH1 and SOD2) were already identified in our previous study as belonging to a common core of up-regulated genes stimulated in fibroblasts by different strains of the same species *B*. *burgdorferi* ss (strain N40 isolated from tick, strains Pbre and 1408 isolated from patients affected by EM or ACA respectively). When focusing on the most highly down-regulated genes, a common core of 9 genes was found to be down-regulated in response to all 3 *Borrelia* species. These genes are mainly involved in the cell division process (Table 4).

**Table 3:** Genes consistently stimulated by the 3 *Borrelia* strains with fold-change values ≥ 2.

Annotation	*B*. *afzelii* (IBS17)		*B*. *garinii* (IBS6)		*B*. *burgdorferi* ss (IBS19)		Description/Function
	Fold change	p-value	Fold change	p-value	Fold change	p-value	
							**Inflammation and innate immune response**
							Chemokines/cytokines
CCL2	5.8	6.20E-04	8.2	2.10E-05	5.2	2.90E-04	Chemoattractant for monocytes and basophils
CXCL1	8.7	3.80E-05	13.4	2.10E-05	7.6	1.90E-03	GROα: chemoattractant for neutrophils
CXCL2	2.8	8.70E-04	3.6	3.30E-04	3.3	1.70E-03	Hematoregulatory chemokine
CXCL6	6	5.10E-04	7.6	1.80E-04	4.7	3.20E-04	Chemoattractant for neutrophils, strong antibacterial activity
CXCL10	6.5	3.90E-02	4.4	9.90E-03	2.5	5.30E-04	Chemoattractant for monocytes and T-lymphocytes
IL6	3.3	2.30E-03	6.2	1.10E-05	2.6	3.90E-03	Cytokine of the acute phase response
IL8	14.9	4.30E-04	20.2	1.90E-05	11	1.00E-03	Chemoattractant for neutrophils
							IFN-related pathway
DDX58	2.2	7.50E-03	2.5	1.60E-03	2.7	4.90E-02	Innate immune receptor, induction of type I IFN and pro-inflammatory cytokines
GBP2	2.1	1.30E-03	2.4	3.80E-03	2.4	6.30E-03	IFN-induced guanylate-binding protein 2: antiviral activity
HERC5	3.2	6.90E-03	3.8	6.00E-04	3.7	1.20E-02	Positive regulator of innate antiviral response in cells induced by IFN
IFI6	2.3	9.10E-03	3.3	1.20E-03	2.6	1.60E-03	IFN-α-inducible protein 6
IFI27	2.7	1.10E-03	5.6	3.60E-05	3.4	3.60E-03	IFN-α-inducible protein 27
IFI44	2.6	2.50E-03	3.2	7.60E-05	2.7	7.40E-03	IFN-induced protein 44
IFI44L	4.3	2.60E-03	7.4	1.90E-04	5.3	4.40E-03	IFN-induced
IFIH1	2.9	2.40E-03	3.3	4.60E-04	3.3	7.60E-03	Induction of type I IFN and pro-inflammatory cytokines
IFIT1	5.2	5.00E-03	5.6	7.00E-05	5.1	1.00E-03	IFN-induced
IFIT2	6.4	1.80E-02	4.8	7.40E-05	4.8	3.00E-03	IFN-induced, activation of IRF by cytosolic pattern recognition receptors
IFIT3	4.4	4.80E-03	5	8.60E-05	4.6	4.80E-03	IFN-induced, inhibition of cell migration and proliferation
IFITM1	2.8	6.70E-04	4.6	9.10E-05	3.5	2.40E-04	IFN-induced transmembrane protein 1
IRF7	2.2	3.10E-03	2.8	9.60E-04	2.8	8.10E-04	IFN regulatory factor 7: transcriptional regulator of type I IFN-dependent immune responses
ISG15	4.4	1.60E-03	6.9	2.10E-04	5.2	5.40E-04	NK cell proliferation, chemoattractant for neutrophils, induce IFN-γ
MX1	8.4	5.10E-04	12.8	7.50E-05	11.1	4.60E-05	IFN-induced GTP-binding protein
MX2	3.5	1.10E-02	4.9	2.50E-04	4.2	3.20E-03	Interferon-induced GTP-binding protein
OAS1	2.9	6.80E-03	3.7	2.00E-04	3.3	3.70E-03	Oligoadenylate synthetase-1: IFN-induced, innate immune response to viral infection
OAS2	4.1	3.00E-03	6	2.00E-04	5.1	4.90E-04	Oligoadenylate synthetase-2: IFN-induced, innate immune response to viral infection
OAS3	2.8	1.10E-03	4.3	1.20E-04	3.5	6.30E-03	Oligoadenylate synthetase-3: IFN-induced, dsRNA-activated antiviral enzyme
RSAD2	4	7.70E-03	4.5	3.40E-04	4.5	2.50E-02	IFN-inducible iron-sulfur cluster-binding antiviral protein
STAT1	2.5	4.60E-04	3.6	5.50E-05	2.9	8.10E-03	Signal transducer of activation-1, up-regulate genes in response to IFN type I, II or III
							NF-kB pathway
NFKBIA	2.2	3.20E-03	2.6	6.30E-04	2.2	3.90E-03	Inhibits the activity of dimeric NF-kappa-B/REL complexes
NFKBIZ	2.9	6.60E-03	3.2	2.50E-03	2.8	1.40E-02	Induction of inflammatory genes activated through TLR/IL-1 receptor signalling
							Complement pathway
CFB	2.7	5.00E-03	4.2	6.30E-04	2.8	2.90E-03	Factor B which is part of the alternate pathway of the complement system
							**Miscellaneous**
C15orf48	2.2	3.90E-04	3.5	1.10E-04	2.5	2.60E-03	Normal mucosa of esophagus-specific gene 1 protein
ECGF1	2	8.20E-03	2.5	2.00E-03	2	2.00E-03	Thymidine phosphorylase: angiogenic activity
EPSTI1	4.4	7.30E-04	6.1	5.40E-05	5.4	8.70E-04	Epithelial-stromal interaction protein 1
HERC6	2.3	5.60E-03	3.3	3.50E-04	2.8	1.60E-02	Probable E3 ubiquitin-protein ligase HERC6
IER3	2.3	3.50E-03	2.5	3.20E-03	2.5	1.10E-02	Role in the ERK signalling pathway
KYNU	2.7	6.20E-03	3.9	3.00E-03	2	2.90E-03	Putative uncharacterized protein KYNU: kynureninase activity
PRIC285	2	4.90E-03	2.7	3.10E-03	2.7	4.60E-03	Helicase with zinc finger domain 2: acts as a transcriptional coactivator for nuclear receptors
SLC39A8	3.3	1.20E-03	4.4	5.70E-04	2.8	3.10E-03	Zinc transporter ZIP8
SOD2	5	8.80E-05	6.7	3.30E-05	4.7	1.60E-04	Superoxide dismutase
TNFAIP6	5.3	3.40E-04	7.8	1.50E-05	4.9	1.20E-03	Cell-cell and cell-matrix interactions
XAF1	2	2.70E-03	2.7	4.90E-04	2.8	6.30E-04	Negative regulator of members of the IAP (Inhibitor of Apoptosis Protein) family

**Table 4:** Genes consistently repressed by the 3 *Borrelia* strains with fold-change values ≤ 0.5.

Annotation	*B*. *afzelii* (IBS 17)		*B*. *garinii* (IBS 6)		*Bb* ss (IBS 19)		Description/Function
	Fold change	p-value	Fold change	p-value	Fold change	p-value	Cell division process
CDC20	0,38	3,97E-06	0,35	1,53E-05	0,41	1,19E-04	Activator of the anaphase promoting complex (APC/C)
PBK	0,50	1,22E-05	0,50	6,95E-05	0,49	5,06E-04	PDZ binding kinase: related to mitogen-activated protein kinase (MAPKK) family
TOP2A	0,46	3,65E-06	0,44	8,88E-06	0,49	3,47E-04	DNA topoisomerase 2-alpha: role in mitosis and meiosis for proper segregation of daughter chromosomes
KIF20A	0,40	3,97E-05	0,37	3,21E-05	0,41	3,15E-05	Mitotic kinesin required for chromosome passenger complex (CPC)-mediated cytokinesis
UBE2C	0,37	1,10E-05	0,36	2,35E-05	0,37	9,67E-05	Ubiquitin-conjugating enzyme E2 C: essential factor of the anaphase promoting complex/cyclosome (APC/C)
CEP55	0,49	1,39E-04	0,47	7,27E-05	0,48	1,72E-03	Centrosomal protein of 55 kDa: role in mitotic exit and cytokinesis
LOC399942	0,44	3,17E-03	0,48	7,97E-03	0,49	2,20E-02	Predicted: similar to Tubulin alpha-2 chain, major constituent of microtubules
ASPM	0,46	8,55E-06	0,44	5,35E-05	0,48	6,88E-05	Probable role in mitotic spindle regulation and coordination of mitotic processes
LOC100132394	0,36	8,45E-04	0,44	8,59E-03	0,36	4,50E-03	Unknown function

### A focus on selected genes among those found to be differentially regulated by microarray analyses

**Fig 5:**
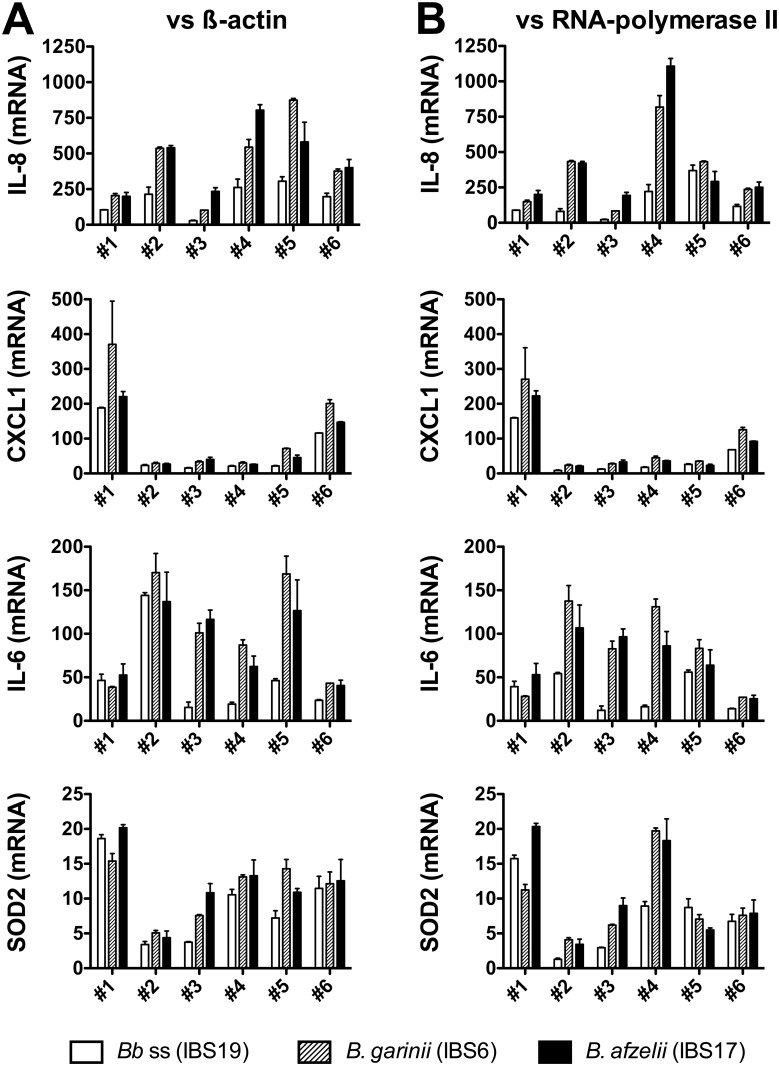
QRT-PCR analysis of mRNA levels induced in human dermal fibroblasts by different species of the *B*. *burgdorferi* sl group. For each batch of fibroblasts,–fibroblasts batch #1 to fibroblasts batch #6 (biological replicates)–, the mRNA levels of IL-8, CXCL1, IL-6 and SOD2 stimulated during 24h at MOI 100:1 by *B*. *burgdorferi* ss IBS19 (white bars), *B*. *garinii* IBS6 (gray hatched bars) and *B*. *afzelii* IBS17 (black bars) were normalized to the β-actin (A) or RNA polymerase II (B) housekeeping gene levels and expressed as relative changes in gene transcription compared with untreated cells. Each bar shows the mean ± SDs of duplicate values (technical replicates).

The mRNA transcription of selected genes found to be up-regulated by microarrays (IL-8, CXCL1, IL-6 and SOD2) was analyzed 24h after *Borrelia* stimulation at MOI 100:1. Using QRT-PCR assays, we confirmed the strong up-regulation of the genes encoding IL-8, CXCL1, IL-6, and SOD2 in all 6 batches of fibroblasts and the three *Borrelia* strains. The QRT-PCR results normalised to the β-actin (Fig 5A) are confirmed by normalising them to the expression of the RNA polymerase II (Fig 5B), another housekeeping gene known to be stable under various stimulatory conditions [20].

**Fig 6:**
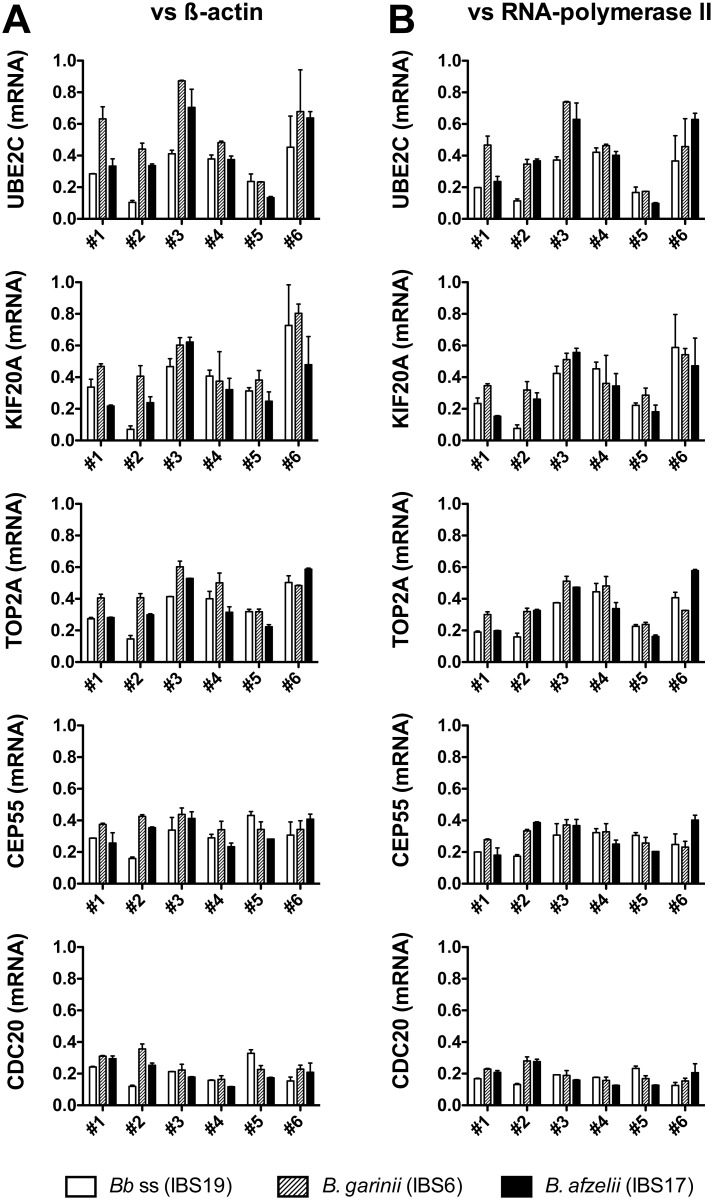
QRT-PCR analysis of mRNA levels repressed in human dermal fibroblasts by different species of the *B*. *burgdorferi* sl group. For each batch of fibroblasts,–fibroblasts batch #1 to fibroblasts batch #6 (biological replicates)–, the mRNA levels of UBE2C, KIF20A, TOP2A, CEP55 and CDC20 stimulated during 24h at MOI 100:1 by *B*. *burgdorferi* ss IBS19 (white bars), *B*. *garinii* IBS6 (gray hatched bars) and *B*. *afzelii* IBS17 (black bars) were normalized to the β-actin (A) or RNA polymerase II (B) housekeeping gene levels and expressed as relative changes in gene transcription compared with untreated cells. Each bar shows the mean ± SDs of duplicate values (technical replicates).

The mRNA transcription of selected genes found to be down-regulated by microarrays (UBE2C, KIF20A, TOP2A, CEP55 and CDC20) was also analyzed under the same experimental conditions. Using QRT-PCR assays, we confirmed a moderate down-regulation of the genes encoding UBE2C, KIF20A, TOP2A, CEP55 and CDC20 in all 6 batches of fibroblasts induced by the three *Borrelia* strains (Fig 6).

## Discussion

Since the 3 strains used in our previous study belonged to the same species, in this new study, we wanted to assess whether the species of *Borrelia* could influence the transcriptional modulations of fibroblasts. For this purpose, we tested 3 *Borrelia* strains, all isolated from erythema migrans lesions and belonging to 3 different pathogenic species (*B*. *afzelii*, *B*. *garinii* and *B*. *burgdorferi* ss) in our NHDF *in vitro* model, and compared the transcriptional profiles induced by the different species using whole transcriptome microarrays. Exploring the interaction between *Borrelia* and fibroblasts, we extended a previous experimental work [19] to a selection of *Borrelia* strains belonging to the three main pathogenic species of *Borrelia* in the North hemisphere, and we increased the range of analyzed transcripts by using a whole genome microarray experiment.

The technical choices of the present study are important. First of all, *Borrelia* strains used were cautiously selected. We tested the most representative *Borrelia* species by selecting one strain of each of the main European *Borrelia* species: *B*. *afzelii*, *B*. *garinii*, and *B*. *burgdorferi* ss [3]. In order to avoid any bias that could be related to the specific environment from which the strains were isolated, we selected 3 clinical *Borrelia* strains that were all isolated from the same type of lesion (EM), the most frequent human clinical manifestation. Bias related to the disseminating potential of *Borrelia* strains was also limited by the selection of strains associated with similar associated clinical features (i.e. without any general symptom suggestive of dissemination associated with EM). All strains tested in our study were cultured in strictly identical conditions, and were all used at low passage (≤P7) in order to avoid the loss of plasmid-encoded virulence factors [22]. The MOI of 100:1 *Borrelia* used for fibroblast stimulations in microarray and QRT-PCR analyses was chosen to be representative of the physiologic spirochete load present in the dermis at the inoculation site a few days after transmission, when the inflammatory process is culminating. Indeed, although only a small number of *Borrelia* are present on tick salivary glands and are then inoculated in the dermis during the feeding process [23, 24], an intense replication of *Borrelia* occurs after inoculation in the host skin at the inoculation site, peaking at day 7 after inoculation [25, 26]. Moreover, setting the stimulation conditions at MOI of 100:1 for 24 hours enabled to yield a potent activation of human dermal fibroblasts while allowing the experimental conditions to emulate those of our first study assessing the early transcriptional response (at 24 hours) of human dermal fibroblasts stimulated by three strains of *B*. *burgdorferi* ss (MOI of 100:1) [19].

Secondly, the microarray experiments were precisely designed and the batches of fibroblasts tested were carefully chosen. A consensual number of 6 different batches of fibroblasts was used in order to reach a sufficient number of biological replicates [27]. Biases were limited by selecting NHDF batches issued from different donors of various sexes and ages, but all batches were issued from areas with similar transcriptional profiles. Indeed, fibroblasts derived from skin at different anatomical sites display distinct transcriptional patterns and this distinction was maintained *in vitro* when fibroblasts were isolated from the influence of other cell types, suggesting that fibroblasts at different locations in the body should be considered distinct differentiated cell types [28, 29]. Approximately 8% of all genes transcribed in fibroblasts were differentially expressed in a site-specific manner and this gene expression signature separates fibroblasts from anterior (rostral) and posterior (caudal) sites of the human body divided by the umbilicus [30]. Thus, in order to avoid the risk of missing transcriptional modifications induced by *Borrelia*, that could be concealed by the heterogeneity of fibroblasts, we chose, in our experiments, fibroblasts issued from a similar transcriptional area (head and chest).

In a previous study, analyzing the interaction between *Borrelia* and fibroblasts, we tested 3 strains of the same species (*B*. *burgdorferi* ss) that were isolated from various clinical environments (strain 1408 isolated from ACA; strain Pbre isolated from EM; strain N40 isolated from a tick). These 3 strains were shown to induce a very similar inflammatory profile in fibroblasts [19]. Therefore, no specific strain-related pathway has been identified that could be linked to the transcriptional responses elicited by clinical manifestations. We thus concluded that *Borrelia* pathotype has little influence on the fibroblasts response. Since the 3 strains used in our previous study belonged to the same species, this new study was designed to determine whether the fibroblasts transcriptional response could be differentially influenced by the *Borrelia* species. The data obtained in this study indicate that the transcriptional response of fibroblasts is largely comparable whichever the *Borrelia* species used for cell stimulation. Indeed, almost all the transcripts with the highest transcriptional modulation were consistently stimulated by the 3 tested species. Transcripts that were specifically modulated by only one strain showed only minor modifications. Thus, our results do not reveal the existence of a fingerprint of transcriptional changes in fibroblasts that could be associated with a particular *Borrelia* species. However, even if the overall *Borrelia*-induced transcriptional modulations in fibroblasts were very similar, an interesting finding of our study was that the level of modification induced by the *B*. *garinii* EM-inducing strain was significantly higher than that of *B*. *afzelii* and *B*. *burgdorferi* ss EM-inducing strains tested. Clinical observations previously reported *B*. *garinii*-induced EM to have shorter incubation and faster evolution than EM caused by *B*. *afzelii* [31, 32]. EM lesions caused by *B*. *garinii* were also significantly larger than EM lesions caused by *B*. *burgdorferi* ss [33] or *B*. *afzelii* [32]. Thus, our *in-vitro* observations are in accordance with the clinical features described by other authors.

Furthermore, the use of a whole genome microarray experiment combined with the analysis of a large number of replicates (3 strains tested with 6 different batches of fibroblasts) allowed an in-depth exploration of the early fibroblasts’ transcriptional response to *Borrelia* infection. The main observation was that the 3 *Borrelia* species induced the stimulation of a large number of pro-inflammatory genes involved in the innate immune response, with high levels of the chemokines/cytokines CCL2, CXCL1, CXCL2, CXCL6, CXCL10, IL-6, IL-8. This result corroborates previous studies reporting the induction of CXCL1, IL6 and IL8 in *Borrelia*-stimulated primary human fibroblasts *in vitro* [19, 34]. *Borrelia*-induced stimulation of dendritic cells and macrophage chemoattractants was also demonstrated in skin biopsies of murine models (MCP-1, also referred to as CCL2) [26], as well as in human biopsies of EM and ACA lesions (CCL2, CXCL1, CXCL9, CXCL10, and CCL20) [35, 36].

We also found that a large number of type I IFN-related genes was intensively stimulated in fibroblasts by *Borrelia*. Type I IFNs play a key role in linking the innate and adaptive immune responses. The stimulation of the type I IFN heteromeric receptor initiates an intracellular cascade leading to the phosphorylation of STAT proteins (especially STAT1) which in turn translocate to the nucleus and initiate transcription by binding specific sites in the promoters of IFN-stimulated genes [37]. The induction of type I IFN by *Borrelia* has been previously shown in human peripheral blood mononuclear cells [38]. A critical role of type I IFN has also been demonstrated in the pathogenesis of *Borrelia* in murine models of Lyme arthritis [39] involving multiple triggering ligands of the bacteria [40]. Fibroblasts were also previously shown to participate in the local propagation of a robust type I IFN response in joint tissues [41]. Since dermal fibroblasts represent the most abundant cells in the site of *Borrelia* inoculation by the tick biting pieces, the strong *Borrelia*-induced stimulation of type I IFN-related genes observed in our experimental model could indicate that fibroblasts actively participate in the initiation of the inflammatory response to *Borrelia* in the skin.

Interestingly, the cytosolic pattern recognition receptor pathway of type 1 IFN induction was also evidenced in fibroblasts stimulated by all 3 studied *Borrelia* strains. This system is triggered by the presence of double-stranded RNA or double-stranded DNA in the cell cytosol following infection by viruses or intracellular bacteria. The cytosolic pattern recognition receptor pathway eventually induces the activation of early phase type 1 IFNs and other proteins implicated in the innate immune response [42]. This finding is interesting since *Borrelia* is classically considered an extracellular pathogen and that only few experimental data currently support that it can be internalised by human dermal fibroblasts [43, 44]. However, considering the complex interplay between pattern recognition receptors and the various cross-regulation of the innate immune receptor signaling [45], this finding should be interpreted with caution since the same IFN-responsive genes and IFN-regulatory factors can be stimulated by pattern recognition receptors in different cellular locations. In the interaction between *Borrelia* and fibroblasts, the transcriptional response of fibroblasts do not seem to be related to the *Borrelia* species tested, since no species-related transcriptional fingerprint could be brought out. Conversely, the fibroblast response appears to be homogeneous whichever the *Borrelia* species or pathotype tested and this cell type seems to play a key role in the inflammatory response elicited by *Borrelia*. The question of the factors involved in the organotropism of Lyme borreliosis manifestations remains unanswered. Factors related to the host are probably more implicated in the pathogenesis of this complex disease and need to be explored in forthcoming studies.

## Supporting Information

S1 Fig Additional titration results of IL-8 secretion by fibroblasts co-incubated with different species of the *B*. *burgdorferi* sl group. Levels of IL-8 secretion by fibroblasts stimulated by increasing concentrations (MOI of 1:1 = 1B, MOI of 10:1 = 10B, 50:1 = 50B, and 100:1 = 100B) of the 3 *Borrelia* strains at 24 hours for fibroblasts batch #3 (A-C) and fibroblasts batch #4 (D-F). NEG: unstimulated fibroblasts. NA: not available data. Each bar shows the mean ± SDs of duplicate values.
(TIF)

S2 Fig Functional analysis of fibroblasts stimulated by *Borrelia*. (A) Fibroblasts stimulated by *B*. *burgdorferi* ss IBS19. (B) Fibroblasts stimulated by *B*. *garinii* IBS6. (C) Fibroblasts stimulated by *B*. *afzelii* IBS17.
(PDF)

S3 Fig Analysis of pathways regulated in fibroblasts by *Borrelia*. (A) Fibroblasts stimulated by *B*. *burgdorferi* ss IBS19. (B) Fibroblasts stimulated by *B*. *garinii* IBS6. (C) Fibroblasts stimulated by *B*. *afzelii* IBS17.
(PDF)

S1 Table Primers used for the quantitative RT-PCR. (PDF)

## Abbreviations

IFN: interferon
EM: erythema migrans
ACA: acrodermatitis chronica atrophicans
NHDF: normal human dermal primary fibroblasts
MOI: multiplicity of infection
SDs: standard deviations
QRT-PCR: semiquantitative reverse transcription PCR
