# Supplementary figures and images for: Homogeneous Inflammatory Gene Profiles Induced in Human Dermal Fibroblasts in Response to the Three Main Species of Borrelia burgdorferi sensu lato
Source: PLoS One. 2016 Oct 5;11(10):e0164117. doi: 10.1371/journal.pone.0164117 (PMC5051687; doi:10.1371/journal.pone.0164117)

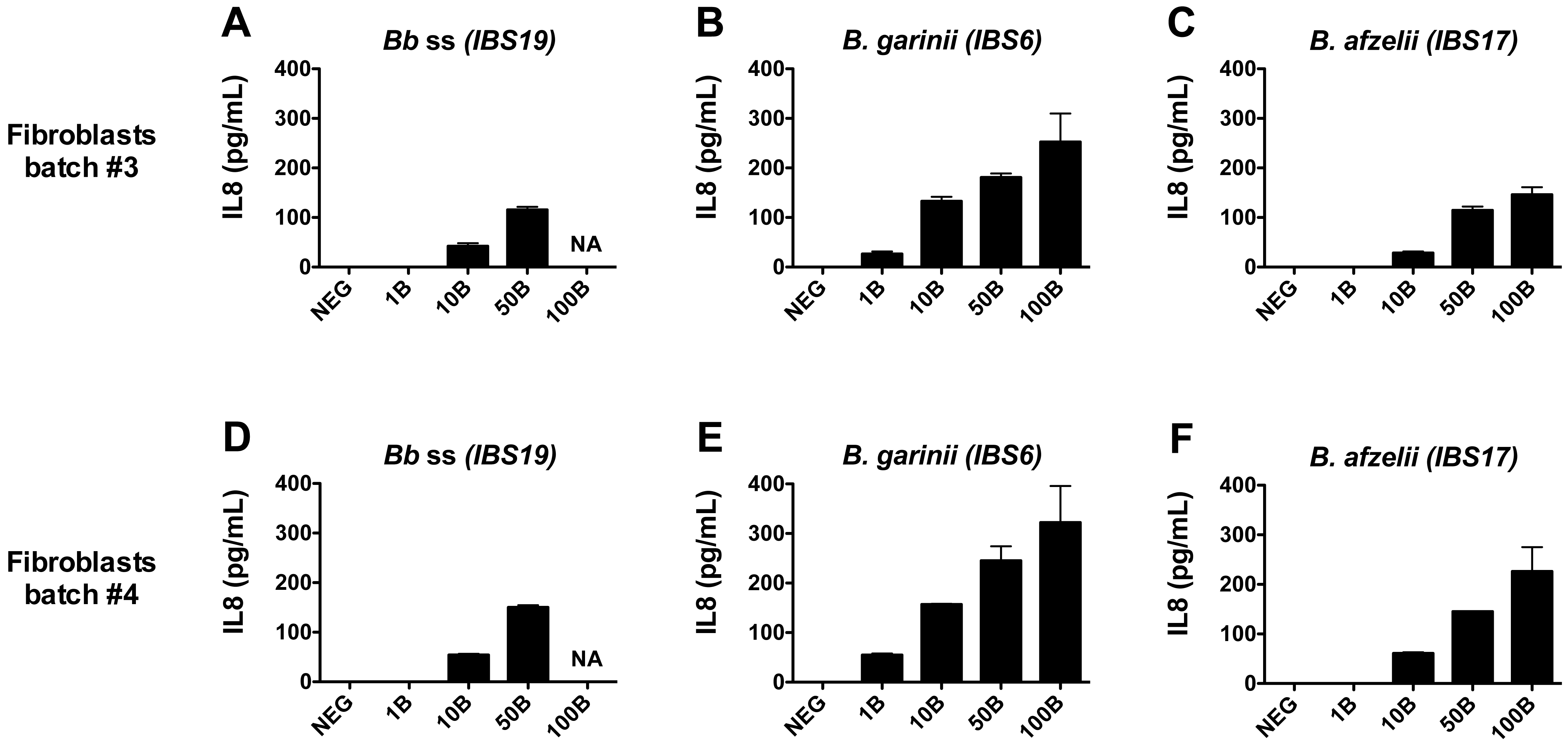

Supplement: S1 Fig — Levels of IL-8 secretion by fibroblasts stimulated by increasing concentrations (MOI of 1:1 = 1B, MOI of 10:1 = 10B, 50:1 = 50B, and 100:1 = 100B) of the 3 Borrelia strains at 24 hours for fibroblasts batch #3 (A-C) and fibroblasts batch #4 (D-F). NEG: unstimulated fibroblasts. NA: not available data. Each bar shows the mean ± SDs of duplicate values. (TIF) [file pone.0164117.s001.tif]
