# Supplementary material for: Homogeneous Inflammatory Gene Profiles Induced in Human Dermal Fibroblasts in Response to the Three Main Species of Borrelia burgdorferi sensu lato
Source: PLoS One. 2016 Oct 5;11(10):e0164117. doi: 10.1371/journal.pone.0164117 (PMC5051687; doi:10.1371/journal.pone.0164117)
Supplement: S2 Fig — (A) Fibroblasts stimulated by B. burgdorferi ss IBS19. (B) Fibroblasts stimulated by B. garinii IBS6. (C) Fibroblasts stimulated by B. afzelii IBS17. (PDF) [file pone.0164117.s002.pdf]

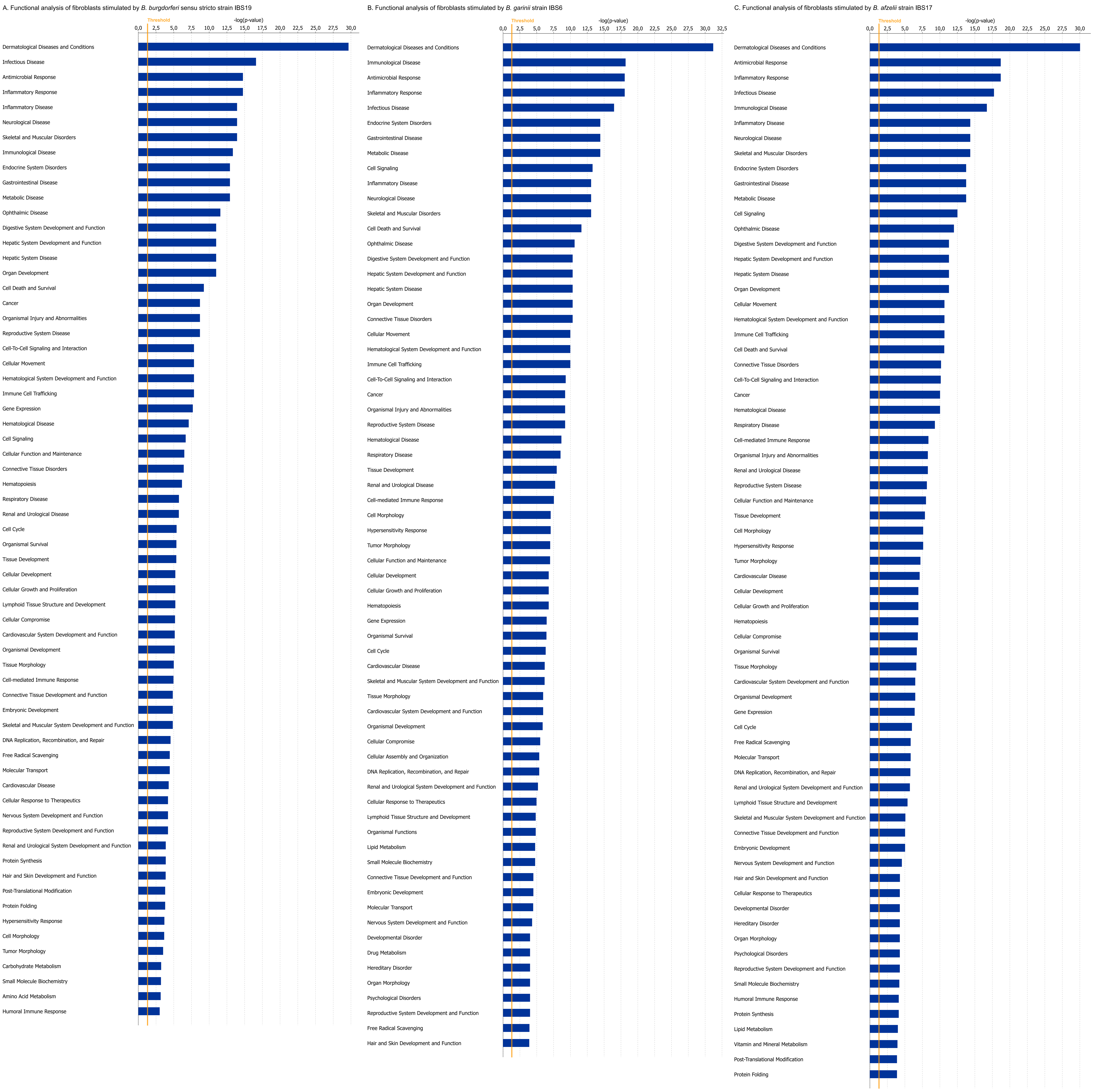
A. Functional analysis of fibroblasts stimulated by B. burgdorferi sensu stricto strain IBS19
Threshold
-log(p-value)
0,0
2,5
5,0
7,5
10,0
12,5
15,0
17,5
20,0
22,5
25,0
27,5
30,0
Dermatological Diseases and Conditions
Infectious Disease
Antimicrobial Response
Inflammatory Response
Inflammatory Disease
Neurological Disease
Skeletal and Muscular Disorders
Immunological Disease
Endocrine System Disorders
Gastrointestinal Disease
Metabolic Disease
Ophthalmic Disease
Digestive System Development and Function
Hepatic System Development and Function
Hepatic System Disease
Organ Development
Cell Death and Survival
Cancer
Organismal Injury and Abnormalities
Reproductive System Disease
Cell-To-Cell Signaling and Interaction
Cellular Movement
Hematological System Development and Function
Immune Cell Trafficking
Gene Expression
Hematological Disease
Cell Signaling
Cellular Function and Maintenance
Connective Tissue Disorders
Hematopoiesis
Respiratory Disease
Renal and Urological Disease
Cell Cycle
Organismal Survival
Tissue Development
Cellular Development
Cellular Growth and Proliferation
Lymphoid Tissue Structure and Development
Cellular Compromise
Cardiovascular System Development and Function
Organismal Development
Tissue Morphology
Cell-mediated Immune Response
Connective Tissue Development and Function
Embryonic Development
Skeletal and Muscular System Development and Function
DNA Replication, Recombination, and Repair
Free Radical Scavenging
Molecular Transport
Cardiovascular Disease
Cellular Response to Therapeutics
Nervous System Development and Function
Reproductive System Development and Function
Renal and Urological System Development and Function
Protein Synthesis
Hair and Skin Development and Function
Post-Translational Modification
Protein Folding
Hypersensitivity Response
Cell Morphology
Tumor Morphology
Carbohydrate Metabolism
Small Molecule Biochemistry
Amino Acid Metabolism
Humoral Immune Response
B. Functional analysis of fibroblasts stimulated by B. garinii strain IBS6
Threshold
-log(p-value)
0,0
2,5
5,0
7,5
10,0
12,5
15,0
17,5
20,0
22,5
25,0
27,5
30,0
32,5
Dermatological Diseases and Conditions
Immunological Disease
Antimicrobial Response
Inflammatory Response
Infectious Disease
Endocrine System Disorders
Gastrointestinal Disease
Metabolic Disease
Cell Signaling
Inflammatory Disease
Neurological Disease
Skeletal and Muscular Disorders
Cell Death and Survival
Ophthalmic Disease
Digestive System Development and Function
Hepatic System Development and Function
Hepatic System Disease
Organ Development
Connective Tissue Disorders
Cellular Movement
Hematological System Development and Function
Immune Cell Trafficking
Cell-To-Cell Signaling and Interaction
Cancer
Organismal Injury and Abnormalities
Reproductive System Disease
Hematological Disease
Respiratory Disease
Tissue Development
Renal and Urological Disease
Cell-mediated Immune Response
Cell Morphology
Hypersensitivity Response
Tumor Morphology
Cellular Function and Maintenance
Cellular Development
Cellular Growth and Proliferation
Hematopoiesis
Gene Expression
Organismal Survival
Cell Cycle
Cardiovascular Disease
Skeletal and Muscular System Development and Function
Tissue Morphology
Cardiovascular System Development and Function
Organismal Development
Cellular Compromise
Cellular Assembly and Organization
DNA Replication, Recombination, and Repair
Renal and Urological System Development and Function
Cellular Response to Therapeutics
Lymphoid Tissue Structure and Development
Organismal Functions
Lipid Metabolism
Small Molecule Biochemistry
Connective Tissue Development and Function
Embryonic Development
Molecular Transport
Nervous System Development and Function
Developmental Disorder
Drug Metabolism
Hereditary Disorder
Organ Morphology
Psychological Disorders
Reproductive System Development and Function
Free Radical Scavenging
Hair and Skin Development and Function
C. Functional analysis of fibroblasts stimulated by B. afzelii strain IBS17
Threshold
-log(p-value)
0,0
2,5
5,0
7,5
10,0
12,5
15,0
17,5
20,0
22,5
25,0
27,5
30,0
Dermatological Diseases and Conditions
Antimicrobial Response
Inflammatory Response
Infectious Disease
Immunological Disease
Inflammatory Disease
Neurological Disease
Skeletal and Muscular Disorders
Endocrine System Disorders
Gastrointestinal Disease
Metabolic Disease
Cell Signaling
Ophthalmic Disease
Digestive System Development and Function
Hepatic System Development and Function
Hepatic System Disease
Organ Development
Cellular Movement
Hematological System Development and Function
Immune Cell Trafficking
Cell Death and Survival
Connective Tissue Disorders
Cell-To-Cell Signaling and Interaction
Cancer
Hematological Disease
Respiratory Disease
Cell-mediated Immune Response
Organismal Injury and Abnormalities
Renal and Urological Disease
Reproductive System Disease
Cellular Function and Maintenance
Tissue Development
Cell Morphology
Hypersensitivity Response
Tumor Morphology
Cardiovascular Disease
Cellular Development
Cellular Growth and Proliferation
Hematopoiesis
Cellular Compromise
Organismal Survival
Tissue Morphology
Cardiovascular System Development and Function
Organismal Development
Gene Expression
Cell Cycle
Free Radical Scavenging
Molecular Transport
DNA Replication, Recombination, and Repair
Renal and Urological System Development and Function
Lymphoid Tissue Structure and Development
Skeletal and Muscular System Development and Function
Connective Tissue Development and Function
Embryonic Development
Nervous System Development and Function
Hair and Skin Development and Function
Cellular Response to Therapeutics
Developmental Disorder
Hereditary Disorder
Organ Morphology
Psychological Disorders
Reproductive System Development and Function
Small Molecule Biochemistry
Humoral Immune Response
Protein Synthesis
Lipid Metabolism
Vitamin and Mineral Metabolism
Post-Translational Modification
Protein Folding
