# Supplementary material for: Homogeneous Inflammatory Gene Profiles Induced in Human Dermal Fibroblasts in Response to the Three Main Species of Borrelia burgdorferi sensu lato
Source: PLoS One. 2016 Oct 5;11(10):e0164117. doi: 10.1371/journal.pone.0164117 (PMC5051687; doi:10.1371/journal.pone.0164117)
Supplement: S3 Fig — (A) Fibroblasts stimulated by B. burgdorferi ss IBS19. (B) Fibroblasts stimulated by B. garinii IBS6. (C) Fibroblasts stimulated by B. afzelii IBS17. (PDF) [file pone.0164117.s003.pdf]

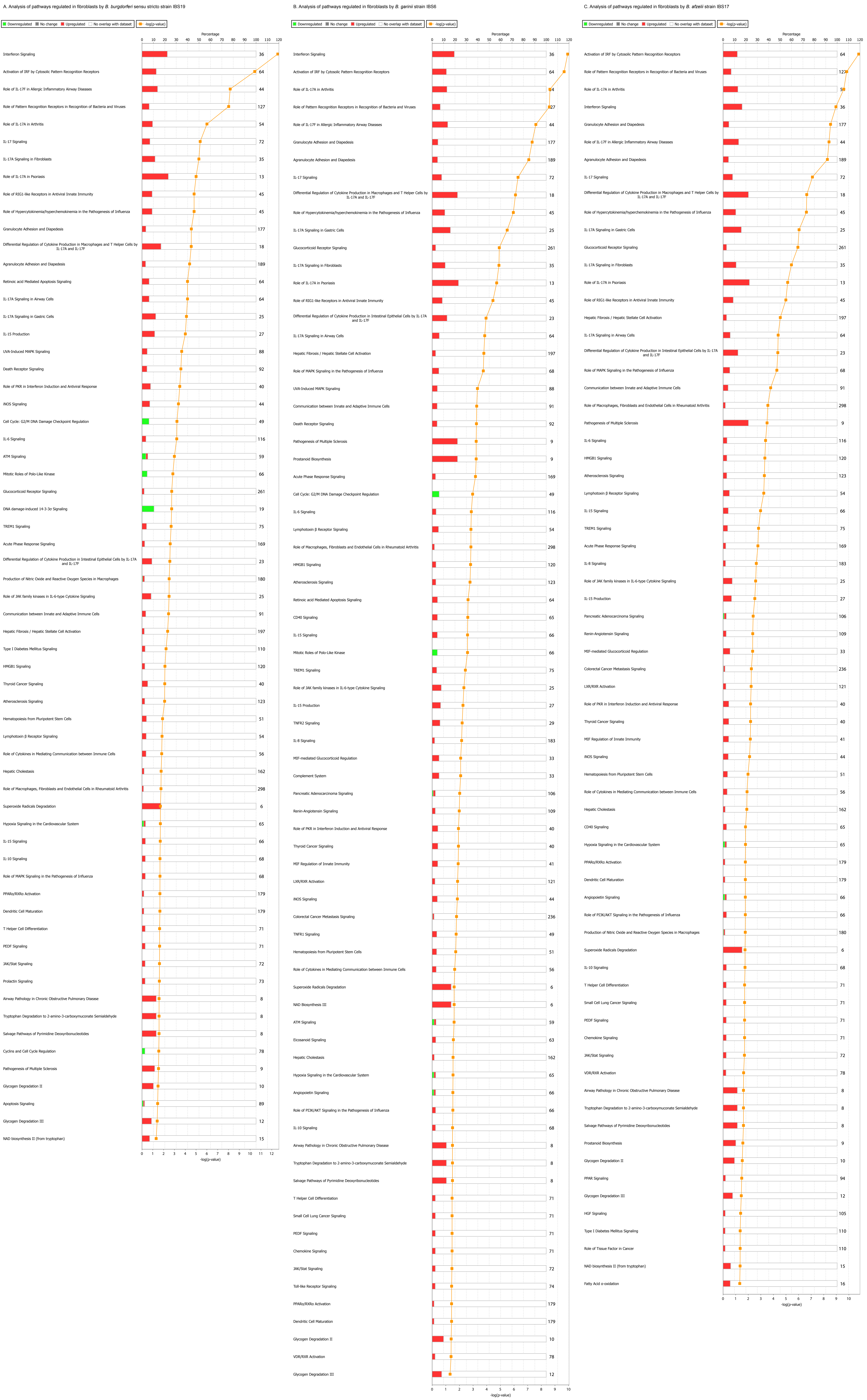
A. Analysis of pathways regulated in fibroblasts by B. burgdorferi sensu stricto strain IBS19
Downregulated
No change
Upregulated
No overlap with dataset
-log(p-value)
Percentage
0 10 20 30 40 50 60 70 80 90 100 110 120
Interferon Signaling 36
Activation of IRF by Cytosolic Pattern Recognition Receptors 64
Role of IL-17F in Allergic Inflammatory Airway Diseases 44
Role of Pattern Recognition Receptors in Recognition of Bacteria and Viruses 127
Role of IL-17A in Arthritis 54
IL-17 Signaling 72
IL-17A Signaling in Fibroblasts 35
Role of IL-17A in Psoriasis 13
Role of RIG1-like Receptors in Antiviral Innate Immunity 45
Role of Hypercytokinemia/hyperchemokinemia in the Pathogenesis of Influenza 45
Granulocyte Adhesion and Diapedesis 177
Differential Regulation of Cytokine Production in Macrophages and T Helper Cells by IL-17A and IL-17F 18
Agranulocyte Adhesion and Diapedesis 189
Retinoic acid Mediated Apoptosis Signaling 64
IL-17A Signaling in Airway Cells 64
IL-17A Signaling in Gastric Cells 25
IL-15 Production 27
UVA-Induced MAPK Signaling 88
Death Receptor Signaling 92
Role of PKR in Interferon Induction and Antiviral Response 40
iNOS Signaling 44
Cell Cycle: G2/M DNA Damage Checkpoint Regulation 49
IL-6 Signaling 116
ATM Signaling 59
Mitotic Roles of Polo-Like Kinase 66
Glucocorticoid Receptor Signaling 261
DNA damage-induced 14-3-3σ Signaling 19
TREM1 Signaling 75
Acute Phase Response Signaling 169
Differential Regulation of Cytokine Production in Intestinal Epithelial Cells by IL-17A and IL-17F 23
Production of Nitric Oxide and Reactive Oxygen Species in Macrophages 180
Role of JAK family kinases in IL-6-type Cytokine Signaling 25
Communication between Innate and Adaptive Immune Cells 91
Hepatic Fibrosis / Hepatic Stellate Cell Activation 197
Type I Diabetes Mellitus Signaling 110
HMGB1 Signaling 120
Thyroid Cancer Signaling 40
Atherosclerosis Signaling 123
Hematopoiesis from Pluripotent Stem Cells 51
Lymphotoxin β Receptor Signaling 54
Role of Cytokines in Mediating Communication between Immune Cells 56
Hepatic Cholestasis 162
Role of Macrophages, Fibroblasts and Endothelial Cells in Rheumatoid Arthritis 298
Superoxide Radicals Degradation 6
Hypoxia Signaling in the Cardiovascular System 65
IL-15 Signaling 66
IL-10 Signaling 68
Role of MAPK Signaling in the Pathogenesis of Influenza 68
PPARα/RXRα Activation 179
Dendritic Cell Maturation 179
T Helper Cell Differentiation 71
PEDF Signaling 71
JAK/Stat Signaling 72
Prolactin Signaling 73
Airway Pathology in Chronic Obstructive Pulmonary Disease 8
Tryptophan Degradation to 2-amino-3-carboxymuconate Semialdehyde 8
Salvage Pathways of Pyrimidine Deoxyribonucleotides 8
Cyclins and Cell Cycle Regulation 78
Pathogenesis of Multiple Sclerosis 9
Glycogen Degradation II 10
Apoptosis Signaling 89
Glycogen Degradation III 12
NAD biosynthesis II (from tryptophan) 15
0 1 2 3 4 5 6 7 8 9 10 11 12
-log(p-value)
B. Analysis of pathways regulated in fibroblasts by B. garinii strain IBS6
Downregulated
No change
Upregulated
No overlap with dataset
-log(p-value)
Percentage
0 10 20 30 40 50 60 70 80 90 100 110 120
Interferon Signaling 36
Activation of IRF by Cytosolic Pattern Recognition Receptors 64
Role of IL-17A in Arthritis 54
Role of Pattern Recognition Receptors in Recognition of Bacteria and Viruses 127
Role of IL-17F in Allergic Inflammatory Airway Diseases 44
Granulocyte Adhesion and Diapedesis 177
Agranulocyte Adhesion and Diapedesis 189
IL-17 Signaling 72
Differential Regulation of Cytokine Production in Macrophages and T Helper Cells by IL-17A and IL-17F 18
Role of Hypercytokinemia/hyperchemokinemia in the Pathogenesis of Influenza 45
IL-17A Signaling in Gastric Cells 25
Glucocorticoid Receptor Signaling 261
IL-17A Signaling in Fibroblasts 35
Role of IL-17A in Psoriasis 13
Role of RIG1-like Receptors in Antiviral Innate Immunity 45
Differential Regulation of Cytokine Production in Intestinal Epithelial Cells by IL-17A and IL-17F 23
IL-17A Signaling in Airway Cells 64
Hepatic Fibrosis / Hepatic Stellate Cell Activation 197
Role of MAPK Signaling in the Pathogenesis of Influenza 68
UVA-Induced MAPK Signaling 88
Communication between Innate and Adaptive Immune Cells 91
Death Receptor Signaling 92
Pathogenesis of Multiple Sclerosis 9
Prostanoid Biosynthesis 9
Acute Phase Response Signaling 169
Cell Cycle: G2/M DNA Damage Checkpoint Regulation 49
IL-6 Signaling 116
Lymphotoxin β Receptor Signaling 54
Role of Macrophages, Fibroblasts and Endothelial Cells in Rheumatoid Arthritis 298
HMGB1 Signaling 120
Atherosclerosis Signaling 123
Retinoic acid Mediated Apoptosis Signaling 64
CD40 Signaling 65
IL-15 Signaling 66
Mitotic Roles of Polo-Like Kinase 66
TREM1 Signaling 75
Role of JAK family kinases in IL-6-type Cytokine Signaling 25
IL-15 Production 27
TNFR2 Signaling 29
IL-8 Signaling 183
MIF-mediated Glucocorticoid Regulation 33
Complement System 33
Pancreatic Adenocarcinoma Signaling 106
Renin-Angiotensin Signaling 109
Role of PKR in Interferon Induction and Antiviral Response 40
Thyroid Cancer Signaling 40
MIF Regulation of Innate Immunity 41
LXR/RXR Activation 121
iNOS Signaling 44
Colorectal Cancer Metastasis Signaling 236
TNFR1 Signaling 49
Hematopoiesis from Pluripotent Stem Cells 51
Role of Cytokines in Mediating Communication between Immune Cells 56
Superoxide Radicals Degradation 6
NAD Biosynthesis III 6
ATM Signaling 59
Eicosanoid Signaling 63
Hepatic Cholestasis 162
Hypoxia Signaling in the Cardiovascular System 65
Angiopoietin Signaling 66
Role of PI3K/AKT Signaling in the Pathogenesis of Influenza 66
IL-10 Signaling 68
Airway Pathology in Chronic Obstructive Pulmonary Disease 8
Tryptophan Degradation to 2-amino-3-carboxymuconate Semialdehyde 8
Salvage Pathways of Pyrimidine Deoxyribonucleotides 8
T Helper Cell Differentiation 71
Small Cell Lung Cancer Signaling 71
PEDF Signaling 71
Chemokine Signaling 71
JAK/Stat Signaling 72
Toll-like Receptor Signaling 74
PPARα/RXRα Activation 179
Dendritic Cell Maturation 179
Glycogen Degradation II 10
VDR/RXR Activation 78
Glycogen Degradation III 12
0 1 2 3 4 5 6 7 8 9 10
-log(p-value)
C. Analysis of pathways regulated in fibroblasts by B. afzelii strain IBS17
Downregulated
No change
Upregulated
No overlap with dataset
-log(p-value)
Percentage
0 10 20 30 40 50 60 70 80 90 100 110 120
Activation of IRF by Cytosolic Pattern Recognition Receptors 64
Role of Pattern Recognition Receptors in Recognition of Bacteria and Viruses 127
Role of IL-17A in Arthritis 54
Interferon Signaling 36
Granulocyte Adhesion and Diapedesis 177
Role of IL-17F in Allergic Inflammatory Airway Diseases 44
Agranulocyte Adhesion and Diapedesis 189
IL-17 Signaling 72
Differential Regulation of Cytokine Production in Macrophages and T Helper Cells by IL-17A and IL-17F 18
Role of Hypercytokinemia/hyperchemokinemia in the Pathogenesis of Influenza 45
IL-17A Signaling in Gastric Cells 25
Glucocorticoid Receptor Signaling 261
IL-17A Signaling in Fibroblasts 35
Role of IL-17A in Psoriasis 13
Role of RIG1-like Receptors in Antiviral Innate Immunity 45
Hepatic Fibrosis / Hepatic Stellate Cell Activation 197
IL-17A Signaling in Airway Cells 64
Differential Regulation of Cytokine Production in Intestinal Epithelial Cells by IL-17A and IL-17F 23
Role of MAPK Signaling in the Pathogenesis of Influenza 68
Communication between Innate and Adaptive Immune Cells 91
Role of Macrophages, Fibroblasts and Endothelial Cells in Rheumatoid Arthritis 298
Pathogenesis of Multiple Sclerosis 9
IL-6 Signaling 116
HMGB1 Signaling 120
Atherosclerosis Signaling 123
Lymphotoxin β Receptor Signaling 54
IL-15 Signaling 66
TREM1 Signaling 75
Acute Phase Response Signaling 169
IL-8 Signaling 183
Role of JAK family kinases in IL-6-type Cytokine Signaling 25
IL-15 Production 27
Pancreatic Adenocarcinoma Signaling 106
Renin-Angiotensin Signaling 109
MIF-mediated Glucocorticoid Regulation 33
Colorectal Cancer Metastasis Signaling 236
LXR/RXR Activation 121
Role of PKR in Interferon Induction and Antiviral Response 40
Thyroid Cancer Signaling 40
MIF Regulation of Innate Immunity 41
iNOS Signaling 44
Hematopoiesis from Pluripotent Stem Cells 51
Role of Cytokines in Mediating Communication between Immune Cells 56
Hepatic Cholestasis 162
CD40 Signaling 65
Hypoxia Signaling in the Cardiovascular System 65
PPARα/RXRα Activation 179
Dendritic Cell Maturation 179
Angiopoietin Signaling 66
Role of PI3K/AKT Signaling in the Pathogenesis of Influenza 66
Production of Nitric Oxide and Reactive Oxygen Species in Macrophages 180
Superoxide Radicals Degradation 6
IL-10 Signaling 68
T Helper Cell Differentiation 71
Small Cell Lung Cancer Signaling 71
PEDF Signaling 71
Chemokine Signaling 71
JAK/Stat Signaling 72
VDR/RXR Activation 78
Airway Pathology in Chronic Obstructive Pulmonary Disease 8
Tryptophan Degradation to 2-amino-3-carboxymuconate Semialdehyde 8
Salvage Pathways of Pyrimidine Deoxyribonucleotides 8
Prostanoid Biosynthesis 9
Glycogen Degradation II 10
PPAR Signaling 94
Glycogen Degradation III 12
HGF Signaling 105
Type I Diabetes Mellitus Signaling 110
Role of Tissue Factor in Cancer 110
NAD biosynthesis II (from tryptophan) 15
Fatty Acid α-oxidation 16
0 1 2 3 4 5 6 7 8 9 10
-log(p-value)
