# Supplementary material for: Homogeneous Inflammatory Gene Profiles Induced in Human Dermal Fibroblasts in Response to the Three Main Species of Borrelia burgdorferi sensu lato
Source: PLoS One. 2016 Oct 5;11(10):e0164117. doi: 10.1371/journal.pone.0164117 (PMC5051687; doi:10.1371/journal.pone.0164117)
Supplement: S1 Table — (PDF) [file pone.0164117.s004.pdf]

S1 Table. Primers used for the quantitative RT-PCR

Primer type		Sequence	Source or reference
Actin	Forward	5’- CCA ACC GCG AGA AGA TGA CC -3’	Schramm et al., PLoS ONE 2012
	Reverse	5’- GAT CTT CAT GAG GTA GTC AGT -3’	PMID: 22768217
RNApol2	Forward	5’- GCA CCA CGT CCA ATG ACA T -3’	Radonic et al., Biochem Biophys Res Commun 2004
	Reverse	5’- GTG CGG CTG CTT CCA TAA -3’	PMID: 14706621
IL-8	Forward	5’- TCT GCA GCT CTG TGT GAA GGT GCA GTT -3’	Marchal et al., Infect Immun 2011
	Reverse	5’- AAC CCT CTG CAC CCA GTT TTC CTT -3’	PMID: 21134970
IL-6	Forward	5’- CCA GAA CAG ATT TGA GAG -3’	Schramm et al., PLoS ONE 2012
	Reverse	5’- CTA CAT TTG CCG AAG AGC -3’	PMID: 22768217
CXCL1	Forward	F : 5’- GTC ACT GTT CAG CAT CTT TTC G -3’	Schramm et al., PLoS ONE 2012
	Reverse	R : 5’- CTG CAT CCC CCA TAG TTA AGA A -3’	PMID: 22768217
MMP12	Forward	F : 5’- TGG CAT TCA GTC CCT GTA TGG AGA -3’	Schramm et al., PLoS ONE 2012
	Reverse	R : 5’- TCC CAC GGT AGT GAC AGC ATC AA -3’	PMID: 22768217
SOD2	Forward	F : 5’- TCG TGG CTG TGG TGG CTT CG -3’	Schramm et al., PLoS ONE 2012
	Reverse	R : 5’- CCT GCT GGT GCC GCA CAC T -3’	PMID: 22768217
UBE2C	Forward	F : 5’- ACC CTC ATG GTA TAT GAA GAC CTG -3’	Designed in this study
	Reverse	R : 5’- AGA GCA GAA TGG TCC TGA CA -3’	
KIF20A	Forward	F : 5’- AGC ACC CTA AAC CAG TTA CCC -3’	Shi et al, Oncotarget 2016
	Reverse	R : 5’- CTG TCG TGG ATT CGC ACT TA -3’	PMID: 27036048
TOP2A	Forward	F : 5’- ACC ATT GCA GCC TGT AAA TGA -3’	Kang et al., Sci Rep 2015
	Reverse	R : 5’- GGG CGG AGC AAA ATA TGT TCC -3’	PMID: 26657567
CEP55	Forward	F : 5’- AGC ACC CTA AAC CAG TTA CCC -3’	Wang et al., Biosci Trends 2016
	Reverse	R : 5’- CTG TCG TGG ATT CGC ACT TA -3’	PMID: 26902787
CDC20	Forward	F : 5’- GTT CGG GTA GCA GAA CAC CA -3’	Designed in this study
	Reverse	R : 5’- CCC CTT GAT GCT GGG TGA AT -3’	
